# Triglyceride-Glucose Index Is Related to Carotid Plaque and Its Stability in Nondiabetic Adults: A Cross-Sectional Study

**DOI:** 10.3389/fneur.2022.823611

**Published:** 2022-03-24

**Authors:** Anran Wang, Yapeng Li, Lue Zhou, Kai Liu, Shaohua Li, Bo Song, Yuan Gao, Yusheng Li, Jie Lu, Chuansheng Tian, Yuming Xu, Longde Wang

**Affiliations:** ^1^Department of Neurology, The First Affiliated Hospital of Zhengzhou University, Zhengzhou, China; ^2^Department of Epidemiology and Health Statistics, College of Public Health, Zhengzhou University, Zhengzhou, China; ^3^Chinese Preventive Medicine Association, Beijing, China; ^4^The General Office of Stroke Prevention Project Committee, National Health Commission of the People's Republic of China, Beijing, China

**Keywords:** carotid plaque, plaque stability, triglyceride-glucose index, insulin resistance, nondiabetic adults

## Abstract

**Background:**

Carotid plaque plays an important role in the development of stroke. The triglyceride-glucose (TyG) index is a reliable alternative marker of insulin resistance. However, there are limited data regarding the relationship between TyG index and carotid plaque and its stability in nondiabetic adults.

**Methods:**

This study was carried out on 24,895 urban workers (10,978 men and 13,917 women) aged 20 years or older who participated in a comprehensive health screening between January 2016 and December 2017 at the First Affiliated Hospital of Zhengzhou University, China. Carotid plaque was assessed using ultrasonography. TyG index was calculated as ln [fasting triglyceride (mg/dL) × fasting glucose (mg/dL) /2]. Logistic regression models and restricted cubic spline (RCS) models were used to estimate the association of the TyG index with carotid plaque and its stability by odds ratios (ORs) and 95% confidence intervals (CIs).

**Results:**

Carotid plaque was detected in 5,668 (22.8%) respondents, with stable and unstable plaque accounting for 2,511 (10.1%) and 3,158 (12.7%), respectively. There was a significant positive association between the prevalence of carotid plaque and TyG index quartile levels, and the same associations were observed for the prevalence of stable and unstable carotid plaque (*P* for trend <0.0001). The multivariable-adjusted ORs (95% CIs) for the highest vs. lowest quartile of TyG index were 1.30 (1.15–1.47) for carotid plaque, 1.38 (1.17–1.63) for stable carotid plaque, and 1.24 (1.07–1.43) for unstable carotid plaque. The RCS analysis showed a linear association between TyG index and carotid plaque, and linear associations were also observed between TyG index and both stable carotid plaque and unstable carotid plaque (*P* for linearity<0.05).

**Conclusion:**

Our findings suggested that the TyG index was significantly associated with carotid plaque and might be a useful indicator for the early identification of carotid plaque in nondiabetic subjects.

## Introduction

Stroke has become a leading cause of disability and mortality worldwide (1). Carotid plaque plays an important role in the development of stroke. Approximately 18–25% of stroke thromboembolisms arise from carotid plaque (2), and carotid plaque was also reported to be an important cause of cryptogenic stroke (3). Rupture of carotid plaque can lead to thrombosis, resulting in vessel occlusion and downstream tissue infarction. A recent systematic review and meta-analysis of the global prevalence of carotid plaque revealed a huge global burden, with 21.1% of people aged 30 to 79 years in 2020 having carotid plaque, equivalent to 815.76 million people (4).

Many studies have shown that early preventive treatment and risk factor intervention are beneficial (5–7). However, carotid ultrasound screening in the general population is not recommended in the current guidelines (8). Therefore, easily accessible and inexpensive biomarkers of carotid plaque can help to identify high-risk individuals who may benefit from early intervention.

Insulin resistance (IR) has been considered as one of the important risk factors for atherosclerosis (9, 10). Studies have shown that the triglyceride-glucose (TyG) index, calculated as ln [fasting triglycerides (mg/dl) × fasting glucose (mg/dl)/2], is a reliable alternative marker of IR compared to the gold standard hyperinsulinemic-glycemic clamp technique (11–13). Several previous studies indicated that the TyG index was associated with atherosclerosis (14, 15), and a growing number of studies have linked the TyG index to cerebro-cardiovascular disease (10, 16–18). However, only a few studies have examined the relationship between TyG index and carotid plaque yielding inconsistent results (15, 19, 20), and most previous studies have focused on whole or diabetic populations, with few studies on nondiabetic populations. Meanwhile, studies on the relationship between TyG index and carotid plaque stability are scarce. Therefore, in the present study, we aim to investigate the relationship of the TyG index with carotid plaque and its stability in nondiabetic adults.

## Methods

### Participants

This study consisted of a population of 28,537 urban workers aged 20 years or older who participated in a health check-up in stroke screening sites of the First Affiliated Hospital of Zhengzhou University from January 2016 to December 2017.

A total of 28,522 participants completed questionnaires, physical examinations, and blood tests. We excluded 3,037 participants with a history of diabetes and 590 subjects with a history of any malignancy, acute inflammation, infectious diseases, or renal disease. Finally, a total of 24,895 participants were enrolled in the current study.

### Data Collection

Individual information on demographic characteristics, personal medical history (hypertension, dyslipidemia, stroke, and coronary heart disease), and lifestyle factors (smoking, drinking, vegetable consumption, fruit consumption, physical activity, etc.) were obtained by trained interviewers using a standard questionnaire. Current smoking was defined as smoking 1 cigarette per day for more than 1 year. Current drinking was defined as consuming at least ≥45 g alcoholic drink each time per day during the last year. Vegetable consumption was divided into two groups (≥5 days/week, <5 days/week) using a daily consumption standard of 200 g of vegetables. Fruit consumption was divided into two groups (≥5 days/week and <5 days/week) using a standard consumption of 200 g of fruit per day. Physical activity was defined as an exercise for at least 30 min per time in no less than 3 times per week.

Physical examinations including height, weight, systolic blood pressure (SBP), and diastolic blood pressure (DBP) were conducted by trained staff. Body mass index (BMI) was calculated as weight in kilograms divided by height in meters squared. Obesity was defined as BMI ≥28 kg/m^2^.

In addition, overnight fasting blood samples were drawn from each participant to test fasting blood glucose (FBG), total cholesterol (TC), triglyceride (TG), high-density lipoprotein cholesterol (HDL-C), and low-density lipoprotein cholesterol (LDL-C). Fasting blood glucose was measured using the glucose oxidase method. Lipid levels including TC, TG, and HDL-C were measured using Olympus Au5400 automated biochemistry analyzer (First Chemical Co, Ltd, Japan) and commercially available kits. LDL-C was calculated by the Friedewald equation when TG was ≤4.5 mmol/L or was directly measured when TG was >4.5 mmol/L. Hypertension was defined as SBP ≥140 mmHg, DBP≥90 mmHg, self-reported hypertension, or the usage of antihypertensive medications. Dyslipidemia was defined as TG ≥2.26 mmol/L, TC ≥6.22 mmol/L, HDL-C <1.04 mmol/L, LDL-C ≥4.14 mmol/L, self-reported diagnosis of dyslipidemia, or taking lipid-lowering drugs.

### Assessment of Carotid Plaque and Its Stability

Carotid plaques were evaluated by qualified sonographers with uniform training, who were unaware of the baseline characteristics and laboratory findings of the participants. Two qualified sonographers measured each participant separately; discrepancies in measurement data were resolved by consensus. We used the iU22 (Philips Healthcare), HA500 (Hitachi Healthcare), and DC-8 (Mindray) ultrasound systems. The transmission frequency of carotid ultrasound images is 5 to 10 MHz. Examination of the bilateral common carotid artery, internal carotid artery, external carotid artery and bulb for the presence of plaque in the transverse and longitudinal directions.

Carotid plaque was defined as a focal structure encroaching into the arterial lumen by at least 0.5 mm or 50% of the surrounding carotid intima–media thickness (CIMT) value, or CIMT > 1.5 mm. Stable carotid plaques were defined as having a homogeneous texture with a regular smooth morphology and high level or homogeneous echogenicity. In contrast, unstable carotid plaques were defined as having an incomplete fibrous cap or ulceration with low level or heterogeneous echogenicity (21).

### Statistical Analysis

The characteristics of the population are presented as frequency (%) for categorical variables and means (standard deviations [SDs]) or median (interquartile ranges [IQRs]) for continuous variables when appropriate. The population was divided into four groups based on the quartiles of the TyG index. Data were compared using chi-square analysis for categorical variables, and analysis of variance (ANOVA) or Mann–Whitney *U*-tests for continuous variables unless otherwise specified.

Logistic regression models were used to investigate the relationship between the TyG index and carotid plaque. To adjust for potential confounders, three models were developed: Model 1, adjusted for age and sex; Model 2, further adjusted for drinking, smoking, vegetable consumption, sports, BMI, hypertension, dyslipidemia, antihypertensive agents, and lipid-lowering agents; and Model 3, Model 2 plus further adjusted for LDL-C, and HDL-C. When participants were divided into 3 categories (no carotid plaque, stable carotid plaque, and unstable carotid plaque), multinomial logistic regression models (Model 1, adjusted for age and sex; Model 2, further adjusted for drinking, smoking, vegetable consumption, sport, BMI, hypertension, dyslipidemia, antihypertensive agents, and lipid-lowering agents; and Model 3, further adjusted for LDL-C, HDL-C) were conducted to adjust for potential confounders. The results are presented as odds ratios (ORs) and 95% confidence intervals (CIs). When the TyG index was treated as a continuous variable, a restricted cubic spline with 3 knots (at the 5th, 50th, and 95th percentiles of the TyG index) was performed to examine the non-linear association between the TyG index and carotid plaque.

Additionally, sensitivity analyses were performed by excluding participants with hypertension, and dyslipidemia.

All analyses above were conducted using R software (version 3.6.3). A two-sided *P* < 0.05 was considered statistically significant.

## Results

### Baseline Characteristics

Among the 24,895 participants, 10,978 (41.1%) participants were men and 13,917 (58.9%) were women. The median (IQR) age of overall participants was 45 (38–52) years, and the median TyG index was 8.50 (IQR 8.15–8.90). Compared with participants in the lowest quartile, those with higher TyG index were more likely to be older and male; to be current smokers, current drinkers, vegetable consumption, fruit consumption, active physical activity, obese, and to have a higher prevalence of dyslipidemia, hypertension, stroke, coronary heart disease, more likely to take antihypertensive agents and lipid-lowering agents, to have a higher level of SBP, DBP, FBG, TC, TG, LDL -C, and more likely to have a lower level of HDL-C. The characteristics of participants according to quartiles of the TyG index are presented in Table 1.

**Table 1:** Baseline characteristics of the study participants.

**Characteristics**	**Overall**	**Quartiles of the TyG index**				***P*-value^†^**
		**Q1 (<8.15)**	**Q2 (8.16–8.50)**	**Q3 (8.51–8.90)**	**Q4 (>8.91)**	
No. of patients	24,895	6,224	6,223	6,227	6,221	
Age, years	46.0 (40.0–54.0)	43.0 (38.0–49.0)	46.0 (40.0–53.0)	48.0 (42.0–55.0)	48.0 (42.0–55.0)	<0.001
Male, sex	10,978 (44.1)	1,39 (27.9)	2,434 (39.1)	2,979 (47.8)	3,826 (61.5)	<0.001
High school or above, n (%)	16,140 (64.8)	4,518(72.6)	4,094 (65.8)	3,843 (61.7)	3,685 (59.2)	<0.001
Smoking, n (%)	5,502 (22.1)	795 (12.8)	1,131 (18.2)	1,506 (24.2)	2,070 (33.3)	<0.001
Drinking, n (%)	4,604 (18.5)	612 (9.8)	936 (15.0)	1,246 (20.0)	1,810 (29.1)	<0.001
Vegetable (<5d/w), n (%)	11,612 (46.6)	2,692 (43.3)	2,878 (46.3)	2,982 (47.9)	3,060 (49.2)	<0.001
Fruit (<5d/w), n (%)	20,396 (81.9)	4,896 (78.7)	5,054 (81.3)	5,182 (83.3)	5,264 (84.7)	<0.001
Active physical activity, n (%)	17,745 (71.3)	4,316 (69.4)	4,482 (72.1)	4,467 (71.8)	4,480 (72.0)	0.001
BMI ≥28 (kg/m^2^)	3,949 (15.9)	397 (6.4)	692 (11.1)	1,165 (18.7)	1,695 (27.2)	<0.001
Hypertension, n (%)	9,279 (37.3)	1,293 (20.8)	2,035 (32.7)	2,678 (43.0)	3,273 (52.6)	<0.001
Dyslipidemia, n (%)	8,626 (34.6)	538 (8.6)	1,180 (19.0)	2,154 (34.6)	4,754 (76.4)	<0.001
Antihypertensive agents, n (%)	2,683 (10.8)	301 (4.8)	547 (8.8)	794 (12.8)	1,041 (16.7)	<0.001
Lipid-lowering agents, n (%)	507 (2.0)	36 (0.6)	82 (1.3)	142 (2.3)	247 (4.0)	<0.001
SBP, mm Hg	128.0 (115.0–144)	119.0 (110.0–132.0)	126.0 (114.0–141.0)	131.0 (118.0–147.0)	136.0 (123.0–151.0)	<0.001
DBP, mm Hg	81.0 (73.0–89.0)	76.0 (69.0–84.0)	79.0 (72.0–88.0)	82.0 (74.0–91.0)	85.0 (77.0–94.0)	<0.001
Fasting blood glucose, mmol/L	5.1 (4.7–5.5)	4.9 (4.6–5.2)	5.1 (4.7–5.4)	5.2 (4.8–5.5)	5.4 (5.0–5.8)	<0.001
Total cholesterol, mmol/L	4.6 (4.1–5.2)	4.3 (3.8–4.7)	4.5 (4.0–5.0)	4.8 (4.2–5.3)	5.0 (4.4–5.6)	<0.001
Triglyceride, mmol/L	1.2 (0.9–1.8)	0.7 (0.6–0.8)	1.0 (0.9–1.1)	1.5 (1.3–1.6)	2.5 (2.0–2.9)	<0.001
HDL cholesterol, mmol/L	1.2 (1.0–1.4)	1.4 (1.2–1.6)	1.3 (1.1–1.5)	1.2 (1.0–1.3)	1.1 (0.9–1.2)	<0.001
LDL cholesterol, mmol/L	2.7 (2.2–3.2)	2.5 (2.1–2.9)	2.8 (2.3–3.2)	2.9 (2.4–3.4)	2.8 (2.2–3.3)	<0.001
Stroke	335 (1.3)	55 (0.9)	87 (1.4)	103 (1.7)	90 (1.4)	0.002
Coronary heart disease	438 (1.8)	70 (1.1)	108 (1.7)	123 (2.0)	137 (2.2)	<0.001

*BMI, body mass index*.

*Continuous variables are expressed as mean ± SD or as median (interquartile range). Categorical variables are expressed as frequencies (percentage)*.

† *P-values were derived from Mann-Whitney U tests for continuous variables, and Chi-square tests for categorical variables*.

**Figure 1:**
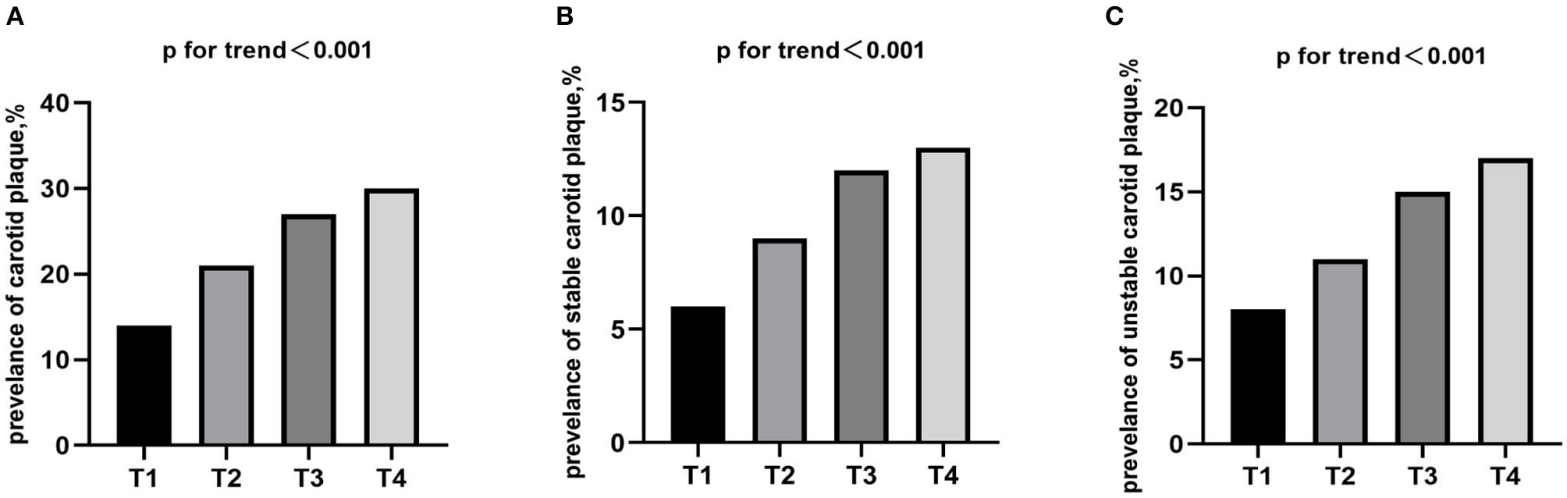
Prevalence of **(A)** carotid plaque, **(B)** stable and **(C)** unstable stratified by quartiles of the TyG index. TyG index, triglyceride-glucose index.

Carotid plaque was detected in 5,668 (22.8%) respondents, with stable and unstable plaque accounting for 2,511 (10.1%) and 3,158 (12.7%), respectively. There was a significant positive association between the prevalence of carotid plaque and TyG index quartile levels, and the same trend was observed for the prevalence of stable and unstable carotid plaque (*P* for trend <0.0001; Figure 1).

### TyG Index and Carotid Plaque

**Figure 2:**
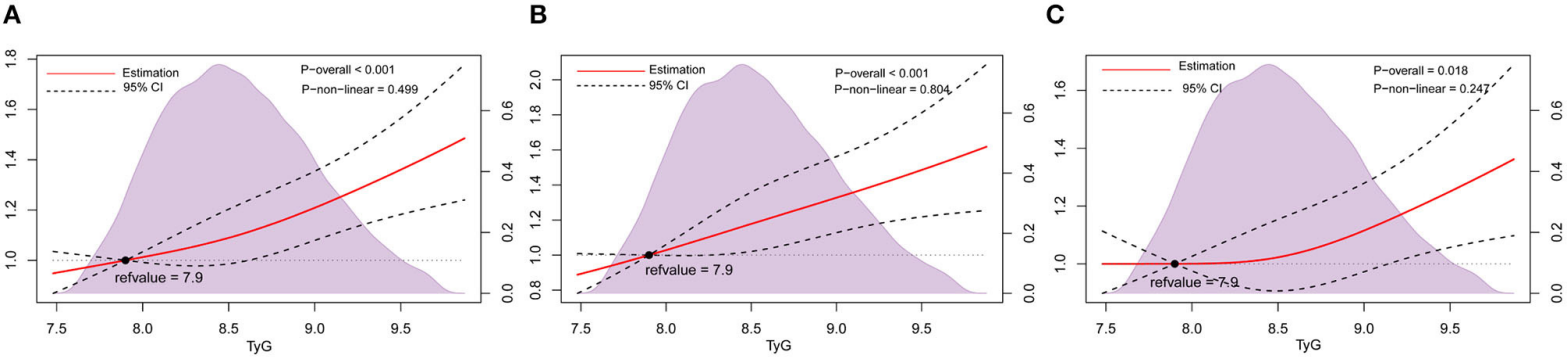
Association of TyG index with carotid plaque and its stability among nondiabetic adults. Odds ratios and 95% CIs derived from restricted cubic spline regression, with knots placed at 5th, 50th, and 95th percentiles of the distribution of TyG index. The reference point for the TyG index is the midpoint (7.9) of the reference group from the categorical analysis. Odds ratios were adjusted for the same variables as Model 3 in Table 2. **(A)**, Carotid plaque. **(B)** Stable carotid plaque. **(C)** Unstable carotid plaque. TyG index, triglyceride-glucose index.

Data were categorized by no carotid plaque, or carotid plaque. The multivariate-adjusted logistic regression models showed that TyG index levels were positively associated with carotid plaque. In model 3, the adjusted ORs (95% CIs) were 1.07 (0.95–1.19) for the second quartile, 1.16 (1.03–1.30) for the third quartile, and 1.30 (1.15–1.47) for the highest quartile compared with the lowest quartile of TyG index. When treated as a continuous variable, every 1 unit increase in the TyG index was associated with a 22% increased prevalent risk of carotid plaque (Table 2). Restricted cubic splines showed a similar trend (Figure 2A).

**Table 2:** Multivariate-adjusted ORs (95% CI) of the association of triglyceride-glucose index with carotid plaque and its stability.

**Outcomes**	**Quartiles of the TyG index**				***P*-trend**	**Per 1**
	**Q1 (<8.15)**	**Q2 (8.16–8.50)**	**Q3 (8.51–8.90)**	**Q4 (>8.91)**		**unit increase**
**Carotid plaque**					
Model 1	Reference	1.25 (1.12–1.40)	1.52 (1.36–1.68)	1.76 (1.59–1.95)	<0.001	1.46 (1.37–1.56)
Model 2	Reference	1.18 (1.06–1.32)	1.35 (1.21–1.51)	1.44 (1.28–1.63)	<0.001	1.26 (1.17–1.37)
Model 3	Reference	1.07 (0.95–1.19)	1.16 (1.03–1.30)	1.30 (1.15–1.47)	<0.001	1.22 (1.13–1.33)
**Stable carotid plaque**					
Model 1	Reference	1.28 (1.10–1.49)	1.54 (1.34–1.78)	1.84 (1.59–2.12)	<0.001	1.53 (1.40–1.67)
Model 2	Reference	1.19 (1.02–1.39)	1.34 (1.15–1.55)	1.43 (1.22–1.68)	<0.001	1.28 (1.15–1.43)
Model 3	Reference	1.12 (0.96–1.31)	1.23 (1.05–1.43)	1.38 (1.17–1.63)	<0.001	1.29 (1.15–1.44)
**Unstable carotid plaque**					
Model 1	Reference	1.23 (1.07–1.40)	1.49 (1.31–1.68)	1.69 (1.50–1.91)	<0.001	1.41 (1.30–1.52)
Model 2	Reference	1.17 (1.03–1.34)	1.35 (1.19–1.54)	1.44 (1.25–1.66)	<0.001	1.25 (1.13–1.37)
Model 3	Reference	1.02 (0.90–1.17)	1.11 (0.96–1.27)	1.24 (1.07–1.43)	0.001	1.17 (1.06–1.30)

*Model 1: adjusted for age, sex*.

*Model 2: adjusted for age, sex, education, smoking status, drinking status, vegetable consumption, fruit consumption, physical activity, BMI ≥28 kg/m^2^ (yes or no), stroke, coronary heart disease, hypertension, dyslipidemia, antihypertensive agents, lipid-lowering agents*.

*Model 3: further adjusted for HDL-C, LDL-C*.

### TyG Index and the Stability of Carotid Plaque

Data were further classified by no carotid plaque, stable carotid plaque and unstable carotid plaque. In multinomial logistic regression models, there was a statistically significant difference between stable carotid plaques and no carotid plaque, and the adjusted ORs (95% CIs) in model 3 were 1.12 (0.96–1.31) for the second quartile, 1.23 (1.05–1.43) for the third quartile, and 1.38 (1.17–1.63) for highest quartile compared with the lowest quartile of TyG index. Furthermore, for every 1 unit increase in the TyG index, the prevalence of stable carotid plaque increased by 1.30 times (Table 2). Restricted cubic splines adjusted for multiple variables showed a similar trend (Figure 2B).

In model 3 compared to the lowest quartile, only the highest quartile showed a statisticant difference between unstable carotid plaque and no carotid plaque, and the adjusted ORs (95% CIs) were 1.02 (0.90–1.17) for the second quartile, 1.11 (0.96–1.27) for the third quartile, 1.24 (1.07–1.43) for the highest quartile compared to the lowest quartile of TyG index. When the TyG index was considered a continuous variable, the prevalence of unstable carotid plaque increased by 1.17 times for every 1 unit increase in the TyG index (Table 2). Restricted cubic splines adjusted for multiple variables showed a similar trend (Figure 2C).

### Sensitivity Analysis

**Figure 3:**
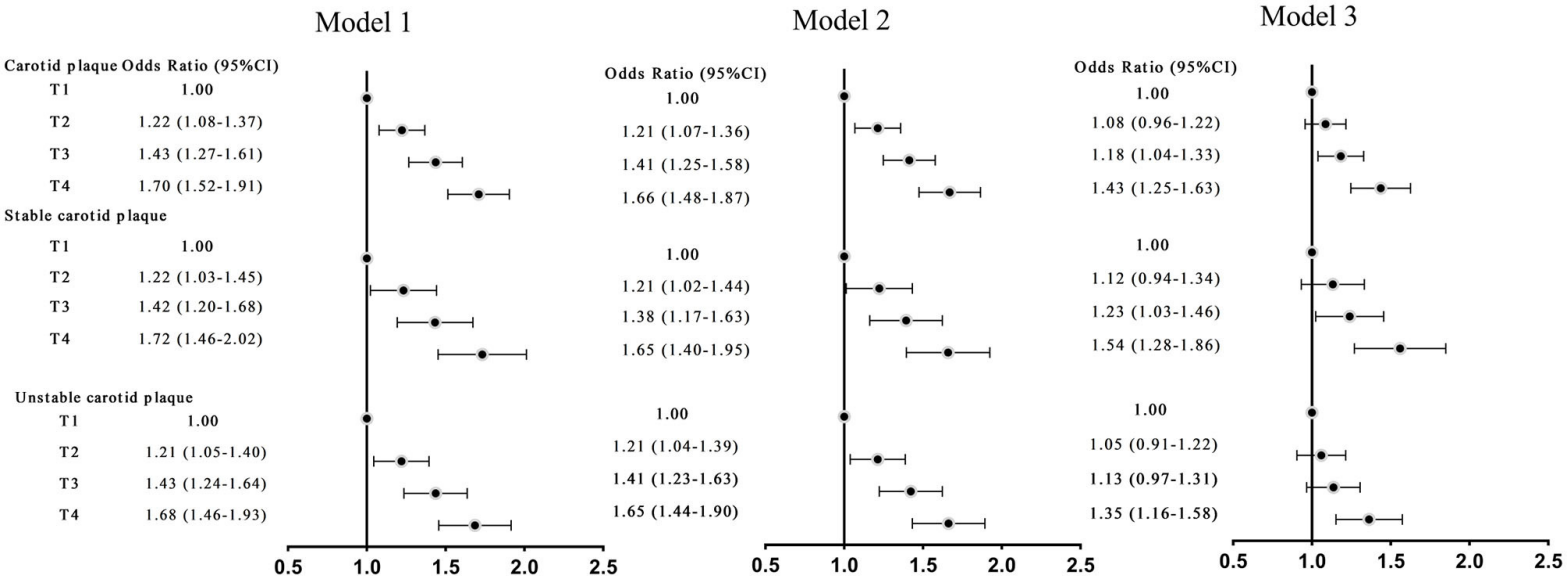
Odds ratios (ORs; 95% CIs) of TyG index and carotid plaque and its stability in adults without hypertension, dyslipidemia. Model 1: adjusted for age, sex. Model 2: adjusted for age, sex, education, smoking status, drinking status, vegetable consumption, fruit consumption, physical activity, BMI ≥28 kg/m^2^ (yes or no), stroke, coronary heart disease. Model 3: further adjusted for HDL-C, LDL-C.

Sensitivity analyses were performed to assess the relationship between TyG and carotid plaque by excluding participants using lipid-lowering drugs, antihypertensive medications, dyslipidemia and hypertension. After multivariable adjustment for the risk factors, sensitivity analyses showed similar results (Figure 3).

## Discussion

This population-based study provided evidence on the association of the TyG index with carotid plaque. We found that a higher TyG index was significantly associated with carotid plaque and this relationship was observed in both stable and unstable carotid plaques. In addition, when the TyG index was used as a continuous variable, linear relationships were observed between the TyG index and carotid plaque, independent of other factors.

Plaques in the carotid arteries have been used as an indicator of systemic vascular atherosclerosis (22). The carotid area is easily accessible for non-invasive studies and is relatively accurate. Carotid plaque plays a major role in the occurrence of cerebrocardiovascular disease (23, 24).

Several previous studies indirectly supported our findings that the TyG index was associated with markers of atherosclerosis. For example, a study based on a healthy Korean population showed that TyG index was associated with increased brachial-ankle pulse wave velocity (baPWV), independent of HDL-C, LDL-C, and other risk factors for atherosclerosis (14). Similarly, a cross-sectional analysis of 4,718 Chinese adults with hypertension also found an independent positive association between the TyG index and baPWV (25). In addition, another study of postmenopausal women in Europe showed that the TyG index was associated with baPWV in lean participants (BMI ≤25 kg/m^2^) (26). Moreover, a recent cohort study found that a higher TyG index was more likely to lead to a higher risk of arterial stiffness (27). Furthermore, a growing number of studies have demonstrated that the TyG index is associated with an increased risk of recurrence, progression and all-cause mortality in patients with cerebrocardiovascular disease (10, 16–18, 28).

However, to date, only a few studies have examined the relationship between the TyG index and carotid plaque and the results remain inconsistent (15, 19, 20). Zhao et al. found that the TyG index was associated with a high risk of atherosclerosis, but not carotid plaque (15). Considering that the participants in the study were over 65 years of age and had a high proportion of lipid-lowering and glucose-lowering medications, the association between TyG index and carotid plaque might be masked. The Asymptomatic Polyvascular Abnormalities Community (APAC) study reported that TyG index was associated with unstable carotid plaque, but not with stable carotid plaque (20). In line with our findings, a cohort study showed that the TyG index increased the risk of carotid atherosclerosis (19). However, HDL-C, LDL-C and other blood markers, well-known risk factors for atherosclerosis, were not adjusted for as confounders in the regression models. Our study extended the current literature and demonstrated that the TyG index was independently associated with carotid plaque and its stability (stable carotid plaque, unstable carotid plaque) in nondiabetic adults.

The mechanism underlying the relationship between TyG and carotid plaque is unclear. The TyG index is a reliable alternative marker for IR, which may play an important role in inferring the relationship between TyG and carotid plaque. Many previous studies have demonstrated that IR plays an important role in the development of atherosclerosis and advanced plaque, by promoting increased synthesis and release of lipoproteins, growth and proliferation of vascular smooth muscle cells, and enhanced transport of LDL-C to arterial smooth muscle cells and inflammation (9, 10, 28–30). In addition, IR is associated with a group of metabolic abnormalities known as insulin resistance syndrome. Each component of insulin resistance syndrome is an independent risk factor for atherosclerosis and plaque formation (9, 31).

Our study has some limitations. First, this was a cross-sectional study and no conclusion can be drawn on the causal relationship between TyG and carotid plaque. Second, the study was carried out in a nondiabetic population, and our findings may not extend to other populations. Considering that most diabetic patients are on long-term glucose-lowering medication or insulin, it may have affected the TyG-carotid plaque relationship. Third, our study was a physical examination population, there were no data to calculate the homeostasis model assessment of insulin resistance (HOMA-IR) to explore the relationship between the HOMA-IR and carotid plaque and its stability. Finally, we did not collect data on plaque size, and the relationship of the TyG index with plaque size should be further studied.

## Conclusion

Our findings suggested that the TyG index was significantly associated with carotid plaque and might be a useful indicator for early identification of carotid plaque in nondiabetic subjects.

## Data Availability Statement

The raw data supporting the conclusions of this article will be made available by the authors, without undue reservation.

## Author Contributions

YX and LW designed the research. YaL, LZ, YG, KL, JL, and SL helped with the acquisition and analysis of the data. AW wrote the article. BS, YG, CT, and YuL contributed to the critical revision of the manuscript. All authors read and approved the final manuscript.

## Funding

This work was supported by a grant from the Ministry of Science and Technology of the People's Republic of China (2018YFC1311303).

## Conflict of Interest

The authors declare that the research was conducted in the absence of any commercial or financial relationships that could be construed as a potential conflict of interest.

## Publisher's Note

All claims expressed in this article are solely those of the authors and do not necessarily represent those of their affiliated organizations, or those of the publisher, the editors and the reviewers. Any product that may be evaluated in this article, or claim that may be made by its manufacturer, is not guaranteed or endorsed by the publisher.
